# Full-Range Moment–Curvature Relationships for Beams Made of Low-Hardening Aluminium Alloys

**DOI:** 10.3390/ma17225545

**Published:** 2024-11-13

**Authors:** Aleksander Szwed, Inez Kamińska, Cezary Ajdukiewicz

**Affiliations:** Faculty of Civil Engineering, Warsaw University of Technology, 00-637 Warsaw, Poland; inez.kaminska@pw.edu.pl (I.K.); cezary.ajdukiewicz@pw.edu.pl (C.A.)

**Keywords:** aluminium alloys, stress–strain relationships, moment–curvature relations, nonlinear elasticity, deformation theory of plasticity, constitutive modelling, bending, beams, strain ductility, curvature capacity, rotation capacity

## Abstract

Aluminium alloys are characterised by a rounded stress–strain relationship, with no sharply defined yield point. For example, aluminium alloy grades 6061-T6, 6082-T6, and 7075-T6 exhibit low-hardening response, which is close to linear elastic-linear plastic hardening characteristics. Commonly, the behaviour of aluminium alloys is described by Ramberg–Osgood (RO) one-dimensional constitutive relationship in the format of strain in terms of stress. In the case of low-hardening response, an alternative Richard–Abbott (RA) relationship of stress as a function of strain can be used. Both relations are analytically irreversible, but the RA is more appropriate for use in slender beams theory. In the present study, we use the latter function to derive moment as an explicit function of curvature for the sectional relation of beams. Since the obtained relation is expressed via special functions, we also propose its close approximation, which is more useful for practical purposes. It is uncomplicated and reasonably accurate compared to available models. The predictive capabilities of the new moment–curvature models developed in this article are verified with experimental results available in the literature for beams tested under four-point and three-point bending. In the case of four-point beams, predictions show very good agreement with experiments, while for three-point bending of beams, higher discrepancies are observed.

## 1. Introduction

In analyses of structural elements under certain loadings, one of the issues to deal with is the selection of an appropriate constitutive relationship adequately reflecting behaviour of the considered material. In the case of metallic alloys, besides the linear elastic model, the currently used material models are formulated in the framework of either plastic deformation theory or plastic flow theory [1,2,3]. The deformation theory of plasticity is equivalent to the nonlinear infinitesimal elasticity theory and can be used if the unloading process can be ignored and no significant redistribution of stresses due to permanent strain is expected. On the other hand, the more general plastic flow theory can be applied to calculations of structures under monotonic or cyclic loading. The plastic flow theory leads to a path-dependent material response in which the actual strain depends on the history of previous deformation. Constitutive equations in this theory are given in the incremental form in which the strain rate is determined by the current stress and its rate. According to this theory, the unloading takes place along a line parallel to the initial elastic path, preserving the initial stiffness of material. This is consistent with experimental behaviour of most structural metals. Conversely, in the deformation theory of plasticity, the current state of stress is uniquely determined by the state of strain, and vice versa. The material response is path-independent and the loading and unloading take place along the same nonlinear stress–strain path. Therefore, the deformation theory lacks the physical foundation compared to the plastic flow theory when permanent deformation occurs. Despite this inconsistency, the simpler deformation theory is used in analysis of many engineering problems involving inelastic (or nonlinear elastic) response, for instance in serviceability and limit state design procedures including stability issues, in which the monotonic loading regimes are regarded. In the analysis of buckling, local or global, the deformation theory seems to be more in agreement with experimental results than the plastic flow theory, what is referred to as the “plastic buckling paradox” [4,5,6,7,8]. A more physically justified plastic flow theory generally leads to overestimated predictions of the critical load, whereas the application of the deformation theory of plasticity in buckling analysis delivers results more compatible with experimental data. For this reason, the deformation theory of plasticity is still recommended for practical engineering applications concerning the inelastic buckling of beams, columns, plates and shells [5,6,9]. Therefore, the analytical models presented in this paper pertain to the deformation theory of plasticity, more precisely to the Green-type nonlinear elasticity.

From a whole host of metallic alloys, aluminium alloys have many advantageous mechanical properties, amongst other things, high strength-to-weight ratio, corrosion resistance, and ability to be formed into beams of complex cross-section shapes. Therefore, they are eagerly used in a wide range of structural applications. The base metal complemented by different amounts of alloying elements, such as magnesium, silicon, copper, zinc, nickel, titanium, zirconium, chromium, and iron, forms a wide variety of aluminium alloys with distinct mechanical properties [10]. Their stress–strain behaviour is characterised by a rounded response (a round house type), with two separate sectors [11]. Initially, the response is almost linear elastic followed by plastic hardening, with a smooth knee transition region between them. Moreover, the stress–strain curves in axial tension tests for different grades display different degrees of nonlinearity. The curvature in the knee zone (close to conventional yield limit) and strain hardening vary, depending on different chemical composition and tempers [12]. In this paper, we focus on the heat-treatable 6xxx and 7xxx series aluminium alloys, which due to this process gain their strength and beneficial mechanical characteristics. The outlook on the use of aluminium alloys in structural engineering is summarised in review papers [13,14].

In the course of analysing nonlinear structural systems, the stress–strain relationship must be represented by a mathematical formula. A number of such material models for metallic alloys have been developed over decades, with the simplest being piecewise linear and piecewise nonlinear models. Several piecewise nonlinear (multi-stage) descriptions can be found in the literature, for example: [11,15,16,17,18]. An alternative approach is to regard a smooth analytical function for full strain range, which can fit experimentally obtained stress–strain curves. A widely used and accepted function is the Ramberg–Osgood (RO) one-dimensional constitutive relationship [19], belonging to a group of functions $\varepsilon \left(\sigma \right)$, giving strain explicitly in terms of stress. Another well-known relationship is the Richard–Abbott (RA) model [20]. It expresses stress explicitly in terms of strain $\sigma \left(\varepsilon \right)$. Unfortunately, besides some special cases, both functions are not reversible analytically. A proposal of an approximate inversion of the multi-stage RO function can be found in works [21,22,23]. The lack of analytical inversibility and the multiple stages with distinct mathematical formulas complicate the derivation of moment–curvature relation for beams and obtaining exact solutions to boundary-value problems.

Currently, plastic design of aluminium alloy structures is not permitted in most design codes, except European provisions which provide recommendations for inelastic analysis [24]. Although aluminium alloys have less ductility compared to steel, they may still have sufficient rotational capacity allowing for moment redistribution and application of plastic design. The design framework of aluminium alloy structures is based on limited research work and often it adopts the same principles as their steel counterparts without considering the main differences between the two materials. A number of papers on aluminium alloy structural members [25,26,27,28,29,30,31,32] demonstrated the influence of strain hardening on the ultimate capacity and the necessity for incorporating this dependence in the codes to improve design efficiency. Recently, based on experimental findings, the Continuous Strength Method (CSM) was proposed to include strain hardening into the design procedures of steel beams [31], and then modified to cover aluminium alloy structures [33]. In this approach, a bi-linear stress–strain $\sigma \left(\varepsilon \right)$ material model is used to obtain sectional moment–curvature relationship for two stages of behaviour. Since the model is quite simple, an equivalent, experimentally justified hardening modulus and ultimate strain ductility were proposed. The hardening modulus and strain ductility parameters used in the CSM model are not directly associated with the stress–strain curve. Moreover, the model does not account for curvature in the knee transition region on the stress–strain curve, which has an influence on overall model prediction and determination of moment capacity and especially curvature ductility. To cover this gap, in this paper, we propose two novel moment–curvature relations based on the direct approximation of the stress–strain curve. The first moment–curvature relation is a result of straightforward implementation of the RA constitutive equation. Because of its complexity, we propose the second full-range continuous nonlinear moment–curvature relation, being a close approximation to the first one, which is more suitable for practical design use. To validate the developed constitutive models, we reference the experimental results for beams with stocky cross-sections, which are available in work [29].

The present paper is organised as follows. After this introduction, in Section 2, a RA type stress–strain relationship is defined via elastic energy potential. Determination of four material parameters included in the model definition is presented in Section 3, with comparisons of the model to the experimental curves. Derivation of the first proposed moment–curvature relationship $M\left(\kappa \right)$ for Bernoulli–Euler slender beams with rectangular hollow cross-sections is given in Section 4. Basic features of the obtained relations are discussed. Next, using parameters of the derived model, the second, simpler moment–curvature relation in the format Richard–Abbott function is proposed in Section 5. The conversion between those two relations is established and discussed. Calibration of the moment–curvature relationships via selected four-point bending tests with comparisons is presented in Section 6. Numerical solutions of boundary-value problems of beams in three-point bending are shown in Section 7. Comparison to experimental data is carried out and discussed. The most relevant outcomes and conclusions are summarised in Section 8.

## 2. One-Dimensional Model of Nonlinear Elastic Material

The elastic strain energy of the one-dimensional elasticity model is defined as [34]:

(1) $$W\left(\varepsilon \right)=\frac{1}{2}E\varepsilon^{2}+\left(E_{0}-E\right)\varepsilon^{2}H_{G}\left[\frac{1}{2n},\frac{1}{n},1+\frac{1}{n},-{\left(\frac{\varepsilon^{2}}{\varepsilon_{0}^{2}}\right)}^{n}\right],$$

where $H_{G}\left(a,b,c,z\right)$ is Gauss hypergeometric function, which can be represented as the following power series:

(2) $$H_{G}\left(a,b,c,z\right)=1+\frac{ab}{c}z+\frac{a\left(1+a\right)b\left(1+b\right)}{c\left(1+c\right)}\frac{z^{2}}{2!}+\frac{a\left(1+a\right)\left(2+a\right)b\left(1+b\right)\left(2+b\right)}{c\left(1+c\right)\left(2+c\right)}\frac{z^{3}}{3!}+\ldots$$

The regarded specific energy function is non-negative $W\left(\varepsilon \right)\geq 0$, null only for the natural state $W\left(0\right)=0$, and convex when $W^{\prime \prime }\left(\varepsilon \right)\geq 0$, resulting in the following restrictions for parameters: $E_{0}>0$, $\varepsilon_{0}\geq 0$, $E_{0}\geq E$ and n>1/2. Function in Equation (1) depends on four material parameters: $E_{0}$, E, $\varepsilon_{0}$ and n, which can be determined from the uniaxial tension experimental test. We define two useful stress parameters $\sigma_{Lim}\geq 0$ and $\sigma_{0}\geq 0$ by the following relations: $\sigma_{Lim}=\left(E_{0}-E\right)\varepsilon_{0}$ and $\sigma_{0}=E_{0}\varepsilon_{0}$. Differentiation of function from Equation (1) leads to the one-dimensional constitutive relation of RA type [20]:

(3) $$\sigma \left(\varepsilon \right)=\frac{dW}{d\varepsilon }=E\varepsilon +\frac{\left(E_{0}-E\right)\varepsilon }{\sqrt[{2n}]{1+{\left(\frac{\varepsilon^{2}}{\varepsilon_{0}^{2}}\right)}^{n}}}.$$

When n→∞ relation in Equation (3) can be written on intervals as a piecewise linear relation, where index *L* stands for the initial linear response and subscript *A* for the asymptotic linear hardening:

(4) $$\sigma_{L}\left(\varepsilon \right)=E_{0}\varepsilon \;\mathrm{for}\;-\varepsilon_{0}\leq \varepsilon \leq \varepsilon_{0}\;\mathrm{and}:$$

(5) $$\sigma_{A}\left(\varepsilon \right)=E\varepsilon +\left(E_{0}-E\right)\varepsilon_{0}\frac{\varepsilon }{\sqrt{\varepsilon^{2}}}\;\mathrm{for}\;\left|\varepsilon \right|>\varepsilon_{0}.$$

Relations according to Equation (5) define two skew asymptotes of the stress–strain relation in Equation (3), one for tension and another for compression regions:

(6) $$\sigma_{AT}\left(\varepsilon \right)=E\varepsilon +\sigma_{Lim}\;\mathrm{and}\;\sigma_{AC}\left(\varepsilon \right)=E\varepsilon -\sigma_{Lim}.$$

**Figure 1:**
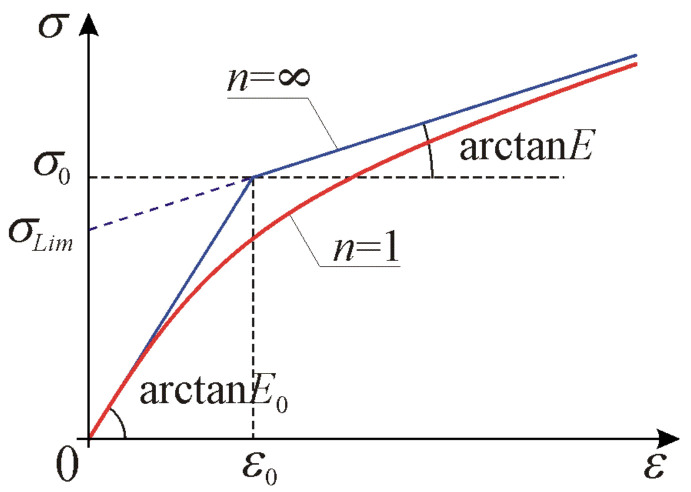
Stress–strain curves according to the one-dimensional model of elastic material for *n* = 1 and n→∞ (piecewise linear). Graphical interpretation of material parameters: *E*_0_, *E*, $\varepsilon_{0}$, $\sigma_{Lim}$ and $\sigma_{0}$.

Graphs of the curve given by Equation (3) for *n* = 1 with line from Equation (4), tangent at the neutral state, and asymptote according to Equation (6) for tension are shown in Figure 1. Exponent *n* can be interpreted as a parameter for smooth regularization of a piecewise linear relationship defined in Equations (4) and (5). All introduced material parameters in the one-dimensional model are explained in Figure 1.

The secant and tangent stiffnesses functions are expressed by formulae:

(7) $$E_{S}\left(\varepsilon \right)=\frac{\sigma }{\varepsilon }=E+\frac{E_{0}-E}{\sqrt[{2n}]{1+{\left(\frac{\varepsilon^{2}}{\varepsilon_{0}^{2}}\right)}^{n}}},\;E_{T}\left(\varepsilon \right)=\frac{d\sigma }{d\varepsilon }=E+\frac{E_{0}-E}{\sqrt[{2n}]{{\left[1+{\left(\frac{\varepsilon^{2}}{\varepsilon_{0}^{2}}\right)}^{n}\right]}^{2n+1}}}.$$

Strict convexity of energy in Equation (1) with respect to strain occurs if $E_{T}\left(\varepsilon \right)>0$ for arbitrary ε. When ε→0 we obtain the initial stiffnesses $E_{S0}=E_{0}$, $E_{T0}=E_{0}$, and for ε→±∞ we obtain the asymptotic stiffnesses values $E_{S\infty }=E$, $E_{T\infty }=E$. Via monotonically increasing function $Q\left(\varepsilon \right)=\sqrt[{2n}]{1+{\left(\varepsilon^{2}/\varepsilon_{0}^{2}\right)}^{n}}$ we are able to control curvature of the knee in the vicinity of point $\left(\varepsilon_{0},\sigma_{0}\right)$ shown in Figure 1.

For low-hardening aluminium alloys we observe a significant difference between the initial and the asymptotic stiffnesses, typically $E_{0}≅300E$, while the regularization parameter takes values in the range n≅1÷6. During calibration of the model parameters, first we estimate the initial modulus of elasticity *E*_0_ and the hardening modulus *E*, then calculate characteristic stress $\sigma_{Lim}$ (or strain $\varepsilon_{0}$), and finally numerically obtain exponent n.

## 3. Determination of Material Parameters

Calibration of the constitutive model parameters is based on the experimental stress–strain curve of uniaxial tension test. Initial elasticity modulus *E*_0_ is estimated from the experimental data located between ten and forty percent of the expected conventional elasticity (yield) limit $\sigma_{Y}$, to avoid anomalies at the beginning of the stress–strain curve and nonlinearity at higher stress levels. Having determined value of *E*_0_ and assuming the plastic strain offset $\varepsilon_{P}=0.002$ the conventional yield limit $\sigma_{Y}$ (i.e., the 0.2% proof stress) with corresponding strain $\varepsilon_{Y}$ are determined. To calculate the strain hardening (asymptotic) modulus, the ultimate stress (strength) $\sigma_{U}$ with corresponding strain $\varepsilon_{U}$ are also captured from the test. For low-hardening aluminium alloy, we assume that $\sigma_{Lim}=\sigma_{Y}$ (see Figure 1), but another representative stress value can be regarded as well. Besides the values of *E*_0_ and $\sigma_{Y}$, the following formulae are used to calculate model parameters $\varepsilon_{Y}$, *E*, $\varepsilon_{0}$ and $\sigma_{Lim}$:

(8) $$\varepsilon_{Y}=\varepsilon_{P}+\frac{\sigma_{Y}}{E_{0}},\;E=\frac{\sigma_{U}-\sigma_{Y}}{\varepsilon_{U}},\;\varepsilon_{0}=\frac{\sigma_{Lim}}{E_{0}-E},\;\sigma_{Lim}=\sigma_{Y}.$$

Finally, the exponent n is calculated as the solution to nonlinear Equation (3) for the yield limit point $\left(\varepsilon_{Y},\sigma_{Y}\right)$.

**Figure 2:**
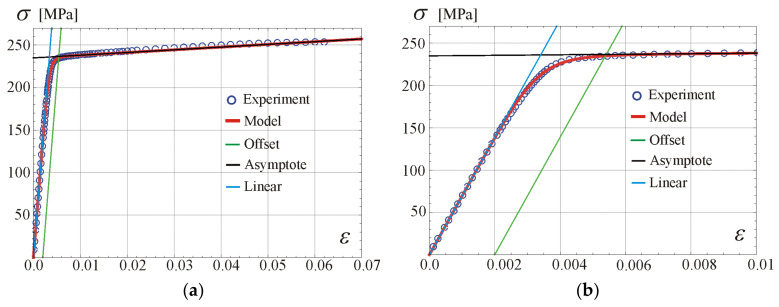

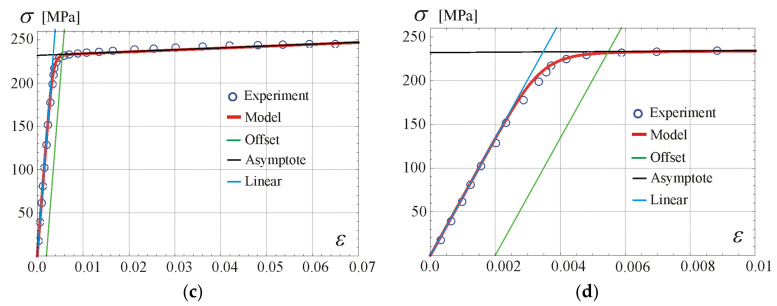
Comparison of the stress–strain relations with experimental data for two aluminium alloys in the whole stable behaviour zone and in the initial strain zone: (**a**,**b**) for 6063-T66 (own experiments), (**c**,**d**) for 6061-T6 [29].

For example, based on our own experimental program, from the carried out uniaxial test of aluminium alloy grade 6063-T66 [34], we estimate $E_{0}=69.3\;\mathrm{GPa}$. The yield stress and strain are: $\sigma_{Y}=235\;\mathrm{MPa}$ and $\varepsilon_{Y}=0.00539$, while the tensile strength is $\sigma_{U}=254\;\mathrm{MPa}$ with corresponding ultimate strain $\varepsilon_{U}=0.06$, hence the hardening ratio is $\sigma_{U}/\sigma_{Y}=1.08$. Next from Equation (8) we obtain: E=318 MPa, $\varepsilon_{0}=0.00341$ and numerically from Equation (3) we obtain n=3.28. The graph of calibrated constitutive relationship in Equation (3) is shown in Figure 2a,b.

In the case of aluminium grade 6061-T6 [29], the values obtained from the stress–strain curve are $\sigma_{Y}=232\;\mathrm{MPa}$, $\varepsilon_{Y}=0.00546$, $\sigma_{U}=245\;\mathrm{MPa}$, $\varepsilon_{U}=0.06$, with the hardening ratio $\sigma_{U}/\sigma_{Y}=1.06$, and the following results for material parameters can be found: $E_{0}=67.0\;\mathrm{GPa}$, E=217 MPa, $\varepsilon_{0}=0.00347$ and n=3.62. Comparison of the calibrated constitutive relationship in Equation (3) with experimental data is shown in Figure 2c,d. Additionally, the offset line 0.2%, the line tangent at the origin, Equation (4), and the asymptote according to Equation (6) with $\sigma_{Lim}=\sigma_{Y}$ are presented. The analytical stress–strain curve from Equation (3) fits very well the experimental points taken from the scan of figure given in [29].

If complete stress–strain curves are not available, for design purposes empirical formulae can be used, compare [11] and [29]:

(9) $$\varepsilon_{U}=0.06+0.1\left(1-\frac{\sigma_{Y}}{\sigma_{U}}\right),\;E=\frac{\sigma_{U}-\sigma_{Y}}{\alpha \varepsilon_{U}-\varepsilon_{Y}},\;\mathrm{with}\;\alpha ≅0.5,$$

where remaining parameters are calculated according to Equation (8). Determination of hardening modulus E according to Equation (9) results in approximately doubled value if compared to the result from Equation (8). Formulae from Equation (9) are recommended by the European design code [24] when aluminium alloy hardening is regarded in designing. They are suitable both for numerical simulations and design methods, particularly in instances where flexure of elements dominates and significant plastic strains are encountered.

## 4. Derivation of Moment–Curvature Relationship for Beams

In this section, using constitutive relationship from Equation (3) for low-hardening aluminium alloys, we derive the moment–curvature relation for a beam cross-section.

Convenient development of analytical moment–curvature relation of slender beams $M\left(\kappa \right)$ requires $\sigma \left(\varepsilon \right)$ format of constitutive relationship, while convenient obtaining solutions to boundary-value problem of a beam needs $\kappa \left(M\right)$ format. Since the Ramberg–Osgood constitutive relationship is a function $\varepsilon \left(\sigma \right)$, then derivation of $M\left(\kappa \right)$ is problematic and requires numerical integration to obtain moment. Mazzolani and Piluso [35] have started with the Ramberg–Osgood type relationship $\kappa \left(M\right)$, which allowed them to obtain analytical solution to some boundary-value problems. In order to establish connection to the constitutive relation $\varepsilon \left(\sigma \right)$ they developed approximate regression formulae based on extensive numerical investigations and experimental data. Conversely, in this work, we propose to use the Richard–Abbott format of the constitutive relationship $\sigma \left(\varepsilon \right)$ for modelling mechanical properties of aluminium alloys. This approach results in a straightforward procedure of obtaining $M\left(\kappa \right)$ sectional relation for beams. Then, having $M\left(\kappa \right)$ a boundary-value problem can be solved via numerical integration of displacement differential equation of equilibrium. Moreover, this format is also convenient for numerical implementation in the finite element method. We propose two novel constitutive functions. The first one is complex but depicts behaviour of aluminium alloys with great precision. The second one is a close approximation of the latter: it is constructed to follow closely the first curve while maintaining simplified mathematical structure.

**Figure 3:**
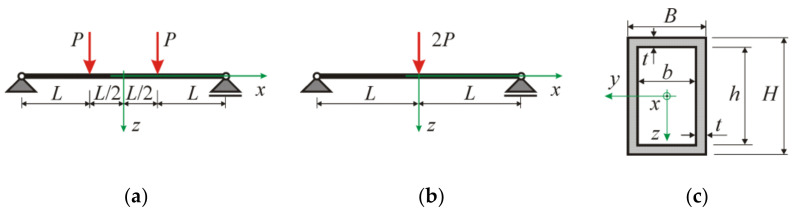
Notation for considered beams and cross-sections: (**a**) four-point bending (B4), (**b**) three-point bending (B3), (**c**) rectangular hollow section of dimensions B×H×t.

Bernoulli’s assumption on the plane cross-section holds. We use the right-handed coordinate system Oxyz shown in Figure 3, where z is the vertical coordinate and x is the longitudinal coordinate. According to the hypothesis, the horizontal displacement u is related to the vertical deflection w of the beam’s neutral axis as:

(10) $$u\left(x,z\right)=z\;\mathrm{tg}\phi \left(x\right),\;\mathrm{where}\;\phi ≅\mathrm{tg}\phi =-\frac{dw\left(x\right)}{dx}.$$

$\phi \left(x\right)$ is the slope function. Then the axial strain ε is related to the curvature $\kappa \left(x\right)$ of the bent beam as:

(11) $$\varepsilon \left(x,z\right)=z\kappa \left(x\right),\;\mathrm{where}\;\kappa \left(x\right)=\frac{d\phi \left(x\right)}{dx}=-\frac{d^{2}w\left(x\right)}{dx^{2}}=-w^{\prime \prime }\left(x\right).$$

Substitution of Equation (11) into Equation (3) leads to the normal stress distribution function in the beam’s cross-section:

(12) $$\sigma \left(x,z\right)=Ez\kappa +\frac{\left(E_{0}-E\right)z\kappa }{\sqrt[{2n}]{1+{\left(\frac{z^{2}\kappa^{2}}{\varepsilon_{0}^{2}}\right)}^{n}}}.$$

Having relation in Equation (12) between the stress and curvature, we obtain bending moment by integration over a cross-section domain. We consider a rectangular hollow section (RHS) B×H×t with external dimensions B×H (width times height) and wall thickness t and the internal dimensions of a hole b×h, where b=B−2t and h=H−2t, compare Figure 3c. Integration leads to the following constitutive relationship for RHS cross-section:

(13) $$\begin{array}{c} M\left(\kappa \right)=\int_{-B/2}^{B/2}\int_{-\;H/2}^{H/2}z\sigma dzdy-\int_{-b/2}^{b/2}\int_{-\;h/2}^{h/2}z\sigma dzdy\\ \;\;\;\;=E\left(J_{R}-J_{H}\right)\kappa +\left(E_{0}-E\right)\kappa \left[J_{R}F_{R}\left(\kappa \right)-J_{H}F_{H}\left(\kappa \right)\right], \end{array}$$

where hypergeometric functions associated with the rectangle $F_{R}$ and the hole $F_{H}$ regions are:

(14) $$\begin{array}{c} F_{R}\left(\kappa \right)=H_{G}\left[\frac{1}{2n},\frac{3}{2n},1+\frac{3}{2n},-{\left(\frac{\kappa^{2}}{\kappa_{E}^{2}}\right)}^{n}\right],\\ F_{H}\left(\kappa \right)=H_{G}\left[\frac{1}{2n},\frac{3}{2n},1+\frac{3}{2n},-{\left(\frac{\chi^{2}\kappa^{2}}{\kappa_{E}^{2}}\right)}^{n}\right]. \end{array}$$

$H_{G}$ is the hypergeometric function given in Equation (2). The following notation is introduced:

(15) $$J_{R}=\frac{BH^{3}}{12},\;J_{H}=\frac{bh^{3}}{12},\;\chi =\frac{h}{H},\;\kappa_{E}=\frac{2}{H}\varepsilon_{0}$$

**Figure 4:**
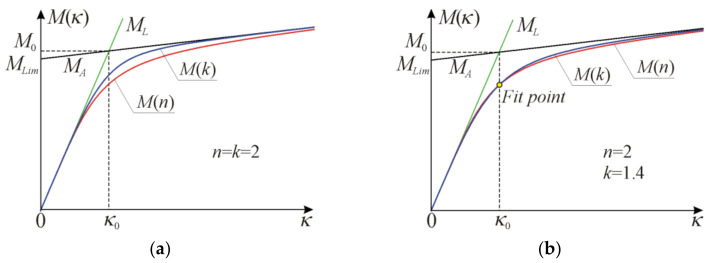
Moment–curvature curves according to Equation (13) ($M\left(n\right)$) and Equation (28) ($M\left(k\right)$) with line tangent at the origin and the asymptote for rectangular cross-section: (**a**) for the same data, (**b**) for fitted *k* exponent.

$J_{R}$ and $J_{H}$ are the second moments of area for the rectangle and the hole, accordingly. Parameter χ we call a section thickness ratio, $\kappa_{E}$ is the characteristic curvature of a bent beam associated with strain $\varepsilon_{0}$. Calculation of the following limit gives the characteristic limit moment $M_{Lim}$ (see Figure 4):

(16) $$M_{Lim}=\underset{\kappa \to \infty }{\lim}\left[M\left(\kappa \right)-E\left(J_{R}-J_{H}\right)\kappa \right]=\frac{3}{2}\kappa_{E}\left(E_{0}-E\right)\left(J_{R}-\frac{J_{H}}{\chi }\right).$$

Tangent stiffness of the cross-section is given by the formula:

(17) $$C_{T}\left(\kappa \right)=E\left(J_{R}-J_{H}\right)+\left(E_{0}-E\right)\left\{\left[\frac{3J_{R}}{Q_{R}\left(\kappa \right)}-\frac{3J_{H}}{Q_{H}\left(\kappa \right)}\right]+2\left[J_{R}F_{R}\left(\kappa \right)-J_{H}F_{H}\left(\kappa \right)\right]\right\},$$

where the curvature dependent functions associated with the rectangle $Q_{R}$ and the hole $Q_{H}$ are:

(18) $$Q_{R}\left(\kappa \right)=\sqrt[{2n}]{1+{\left(\frac{\kappa^{2}}{\kappa_{E}^{2}}\right)}^{n}},\;Q_{H}\left(\kappa \right)=\sqrt[{2n}]{1+{\left(\frac{\chi^{2}\kappa^{2}}{\kappa_{E}^{2}}\right)}^{n}}.$$

Calculation of the following limits results in the initial $C_{0}$ and asymptotic $C_{A}$ stiffnesses of the cross-section:

(19) $$C_{0}=\underset{\kappa \to 0}{\lim}C_{T}\left(\kappa \right)=E_{0}\left(J_{R}-J_{H}\right),\;C_{A}=\underset{\kappa \to \infty }{\lim}C_{T}\left(\kappa \right)=E\left(J_{R}-J_{H}\right).$$

Based on Equation (19), we can define a line tangent at the natural state and, with usage of Equation (16), skew asymptotes:

(20) $$M_{L}\left(\kappa \right)=E_{0}\left(J_{R}-J_{H}\right)\kappa ,\;M_{A}\left(\kappa \right)=\pm M_{Lim}+E\left(J_{R}-J_{H}\right)\kappa .$$

Lines given by Equation (20) intersect for curvature $\kappa_{0}$ and moment $M_{0}$ (see Figure 4):

(21) $$\kappa_{0}=\frac{3}{2}\kappa_{E}\frac{J_{R}\chi -J_{H}}{\left(J_{R}-J_{H}\right)\chi },\;M_{0}=\frac{3}{2}\kappa_{E}E_{0}\left(J_{R}-\frac{J_{H}}{\chi }\right).$$

A typical graph of moment–curvature relation according to Equation (13), denoted as $M\left(n\right)$, and lines described by Equation (20) with interpretation of parameters $M_{Lim}$, $\kappa_{0}$ and $M_{0}$ with n=2 for rectangular cross-section are shown in Figure 4.

By integration of function from Equation (13), we can determine the specific elastic energy for the beam cross-section:

(22) $$\begin{array}{c} W\left(\kappa \right)=\frac{1}{2}E\left(J_{R}-J_{H}\right)\kappa^{2}-\frac{3}{2}\left(E_{0}-E\right)\kappa^{2}\left[J_{R}F_{R1}\left(\kappa \right)-J_{H}F_{H1}\left(\kappa \right)\right]\\ \;\;\;\;+\left(E_{0}-E\right)\kappa^{2}\left[J_{R}F_{R}\left(\kappa \right)+J_{H}F_{H}\left(\kappa \right)\right], \end{array}$$

where additional hypergeometric functions associated with the rectangle $F_{R1}$ and the hole $F_{H1}$ are:

(23) $$F_{R1}\left(\kappa \right)=H_{G}\left[\frac{1}{2n},\frac{1}{n},1+\frac{1}{n},-{\left(\frac{\kappa^{2}}{\kappa_{E}^{2}}\right)}^{n}\right],\;F_{H1}\left(\kappa \right)=H_{G}\left[\frac{1}{2n},\frac{1}{n},1+\frac{1}{n},-{\left(\frac{\chi^{2}\kappa^{2}}{\kappa_{E}^{2}}\right)}^{n}\right].$$

Functions given in Equations (13), (17) and (22) have complex mathematical forms since they are expressed via special hypergeometric functions. This can be a limitation for the practical use of the obtained constitutive relationships. Note that for the special case with *n* = 1 the aforementioned functions can be expressed via elementary functions:

(24) $$\begin{array}{c} W\left(\kappa \right)=\frac{1}{2}E\left(J_{R}-J_{H}\right)\kappa^{2}+\frac{3}{2}\left(E_{0}-E\right)J_{R}\kappa_{E}^{2}\left[\sqrt{1+\frac{\kappa^{2}}{\kappa_{E}^{2}}}+\frac{\kappa_{E}}{\kappa }\mathrm{arsinh}\left(\frac{\kappa }{\kappa_{E}}\right)-2\right]\\ \;\;\;\;\;-\frac{3}{2}\left(E_{0}-E\right)J_{H}\frac{\kappa_{E}^{2}}{\chi^{2}}\left[\sqrt{1+\frac{\chi^{2}\kappa^{2}}{\kappa_{E}^{2}}}+\frac{\kappa_{E}}{\chi \kappa }\mathrm{arsinh}\left(\frac{\chi \kappa }{\kappa_{E}}\right)-2\right], \end{array}$$

(25) $$\begin{array}{c} M\left(\kappa \right)=E\left(J_{R}-J_{H}\right)\kappa +\frac{3}{2}\left(E_{0}-E\right)J_{R}\kappa_{E}\left[\frac{\kappa_{E}}{\kappa }\sqrt{1+\frac{\kappa^{2}}{\kappa_{E}^{2}}}-\frac{\kappa_{E}^{2}}{\kappa^{2}}\mathrm{arsinh}\left(\frac{\kappa }{\kappa_{E}}\right)\right]\\ \;\;\;\;\;-\frac{3}{2}\left(E_{0}-E\right)J_{H}\frac{\kappa_{E}}{\chi }\left[\frac{\kappa_{E}}{\chi \kappa }\sqrt{1+\frac{\chi^{2}\kappa^{2}}{\kappa_{E}^{2}}}-\frac{\kappa_{E}^{2}}{\chi^{2}\kappa^{2}}\mathrm{arsinh}\left(\frac{\chi \kappa }{\kappa_{E}}\right)\right], \end{array}$$

(26) $$\begin{array}{c} C_{T}\left(\kappa \right)=E\left(J_{R}-J_{H}\right)+3\left(E_{0}-E\right)J_{R}\left[\frac{\kappa_{E}^{3}}{\kappa^{3}}\mathrm{arsinh}\left(\frac{\kappa }{\kappa_{E}}\right)-\frac{\frac{\kappa_{E}^{2}}{\kappa^{2}}}{\sqrt{1+\frac{\kappa^{2}}{\kappa_{E}^{2}}}}\right]\\ \;\;\;\;\;-3\left(E_{0}-E\right)J_{H}\left[\frac{\kappa_{E}^{3}}{\chi^{3}\kappa^{3}}\mathrm{arsinh}\left(\frac{\chi \kappa }{\kappa_{E}}\right)-\frac{\frac{\kappa_{E}^{2}}{\chi^{2}\kappa^{2}}}{\sqrt{1+\frac{\chi^{2}\kappa^{2}}{\kappa_{E}^{2}}}}\right]. \end{array}$$

## 5. Proposal of Simplified Moment–Curvature Relationship for Practical Use

The constitutive relationship between moment and curvature given by Equation (13), even detailed in Equation (25), involves complicated mathematical expressions. On the other hand, the moment–curvature relationship in a form similar to Equation (3) seems to be a good choice for practical application. That is why there is a need for the development of a simpler expression while maintaining the basic features of the original relations in Equations (13), (17) and (22). We propose the following specific elasticity energy, constitutive relationship, and tangent stiffness functions for a beam cross-section:

(27) $$W\left(\kappa \right)=\frac{1}{2}E\left(J_{R}-J_{H}\right)\kappa^{2}+\frac{1}{2}\left(E_{0}-E\right)\left(J_{R}-J_{H}\right)\kappa^{2}H_{G}\left[\frac{1}{2k},\frac{1}{k},1+\frac{1}{k},-{\left(\frac{\kappa^{2}}{\kappa_{0}^{2}}\right)}^{k}\right],$$

(28) $$M\left(\kappa \right)=E\left(J_{R}-J_{H}\right)\kappa +\frac{\left(E_{0}-E\right)\left(J_{R}-J_{H}\right)\kappa }{\sqrt[{2k}]{1+{\left(\frac{\kappa^{2}}{\kappa_{0}^{2}}\right)}^{k}}},$$

(29) $$C_{T}\left(\kappa \right)=E\left(J_{R}-J_{H}\right)+\frac{\left(E_{0}-E\right)\left(J_{R}-J_{H}\right)}{\sqrt[{2k}]{{\left[1+{\left(\frac{\kappa^{2}}{\kappa_{0}^{2}}\right)}^{k}\right]}^{2k+1}}}.$$

Note that the basic model characteristics obtained in Equations (16), (19) and (21) are the same, while the exponent n in Equation (12) needs to be adjusted. The relation from Equation (28), denoted as $M\left(k\right)$, and functions from Equation (20) with interpretation of parameters $M_{Lim}$, $\kappa_{0}$ and $M_{0}$ with k=n=2 for rectangular cross-section are shown in Figure 4a, in comparison to the original function given by Equation (13). Note that when the exponents are equalised k=n, a significant discrepancy between curves $M\left(n\right)$ and $M\left(k\right)$ is observed in Figure 4a.

Therefore, a different exponent *k* and the same characteristic curvature $\kappa_{0}$ described by Equation (21) are used in Equations (27)–(29). To have good compatibility between the proposals from Equations (13) and (28) we require the same value of moments for the characteristic curvature $\kappa =\kappa_{0}$, which leads to the following formula for the conversion of exponents:

(30) $$k\left(n\right)=\frac{\ln2}{2\left\{\ln\left(J_{R}-J_{H}\right)-\ln\left[J_{R}H_{GH}\left(n\right)-J_{H}H_{GR}\left(n\right)\right]\right\}},$$

where:

(31) $$H_{GR}=H_{G}\left[\frac{1}{2n},\frac{3}{2n},1+\frac{3}{2n},-{\left(\frac{\kappa_{0}}{\kappa_{E}}\right)}^{2n}\right],\;H_{GH}=H_{G}\left[\frac{1}{2n},\frac{3}{2n},1+\frac{3}{2n},-{\left(\frac{\chi \kappa_{0}}{\kappa_{E}}\right)}^{2n}\right].$$

Note that the conversion exponent $k\left(n\right)$ depends on material properties and cross-sectional parameters. Relation $M\left(k\right)$ given by Equation (28) for a rectangular cross-section with adjustment of k=1.4 for n=2 is shown in Figure 4b. Now, the relative difference between two relations $M\left(n\right)$ and $M\left(k\left(n\right)\right)$ is much smaller if compared to curves shown on Figure 4a. For the investigated case, the maximum difference does not exceed 3%, and as a general trend, the difference increases with increasing *n*. Typically, the value of exponent *k* is smaller than that of *n*. For RHS with dimensions B=95 mm, H=50 mm, t=10.5 mm made of aluminium grade 6061-T6 [29] with material data $E_{0}=67\;\mathrm{GPa}$, E=217 MPa, $\varepsilon_{0}=0.00347$, $\kappa_{E}=0.139\;m^{-1}$, $\kappa_{0}=0.181\;m^{-1}$, the results of conversion according to Equation (30) are shown in Table 1.

## 6. Calibration of Moment–Curvature Relationships Based on Four-Point Bending Tests

To investigate the descriptive capabilities of the presented models, we use the results of experiments carried out in [29] on aluminium beams with rectangular hollow sections, as shown in Figure 3c. To verify the proposed relationships for beams with stocky cross-sections, sections with low relative thickness ratios (b/t or h/t less than 10) were selected. For such RHS of the tested beams, local buckling of plate elements was precluded, which was confirmed by observing failure by material yielding or tensile fracture.

The experimental relationship between moment and curvature was determined from four-point bending tests (B4), as shown in Figure 3a. Measurements of displacements at load application points and midspan allowed the calculation of curvature of a bent beam in the midspan constant moment zone. To calibrate the parameters included in the moment–curvature relationships from Equations (13) and (28), the *FindFit* procedure of *Wolfram Mathematica* v.12.1 software was used. Values of four material parameters *E*_0_, *E*, *ε*_0_, n included in Equation (13) with additional characteristic curvatures $\kappa_{E}$, $\kappa_{0}$ and moment $M_{0}$ are given in Table 2. Values of the conversion exponent $k\left(n\right)$ according to Equation (30) are also presented for four beams made of low-hardening aluminium alloy grade 6061-T6. Table 3 shows the values of material parameters *E*_0_, *E*, $\varepsilon_{0}$, *k* calculated according to Equation (28) with characteristic curvatures $\kappa_{E}$, $\kappa_{0}$ and moment $M_{0}$. A strong correlation between the values of the parameters of the two moment–curvature relationships can be observed. The values of material stiffness parameters *E*_0_ and *E* resulting from Equation (28) are consistently slightly higher than those for Equation (13). In contrast, parameters *ε*_0_ and *k* are lower for the relation in Equation (28) than for the relation in Equation (13) when compared to the converted exponent $k\left(n\right)$.

**Figure 5:**
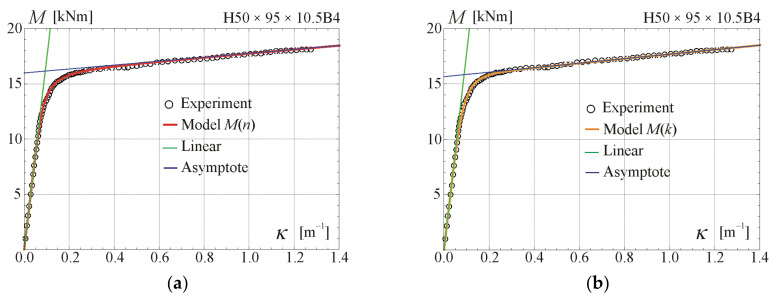
Moment–curvature relations for the four-point bending of beam H50 × 95 × 10.5B4 tested in [29]: (**a**) fitted Equation (13), (**b**) fitted Equation (28).

**Figure 6:**
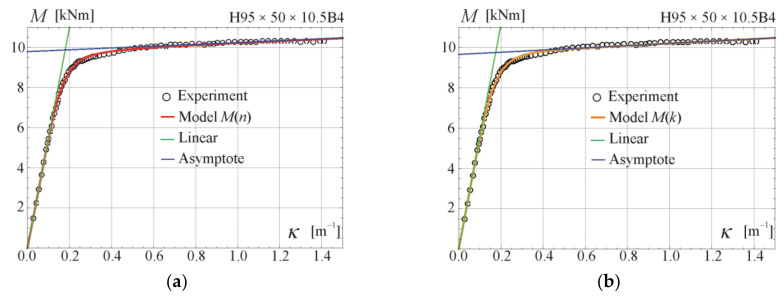
Moment–curvature relations for the four-point bending of beam H95 × 50 × 10.5B4 tested in [29]: (**a**) fitted Equation (13), (**b**) fitted Equation (28).

**Figure 7:**
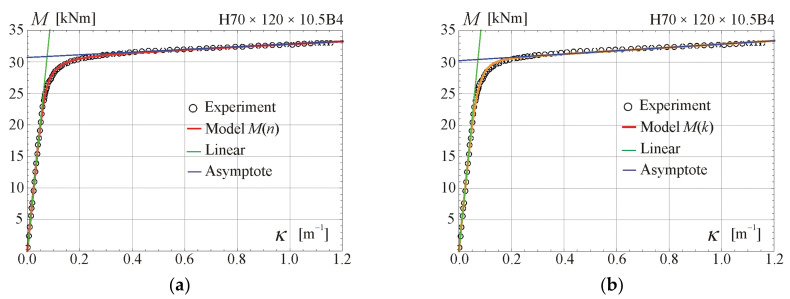
Moment–curvature relations for the four-point bending of beam H70 × 120 × 10.5B4 tested in [29]: (**a**) fitted Equation (13), (**b**) fitted Equation (28).

**Figure 8:**
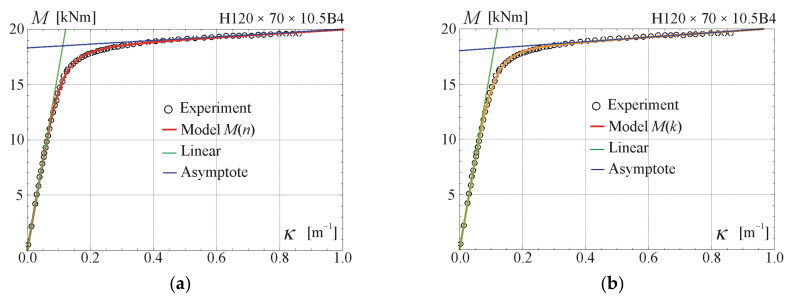
Moment–curvature relations for the four-point bending of beam H120 × 70 × 10.5B4 tested in [29]: (**a**) fitted Equation (13), (**b**) fitted Equation (28).

Comparisons of the moment–curvature curves of the relationships in Equations (13) and (28) with experimental points taken from the scanned figures given in [29] are presented in Figure 5, Figure 6, Figure 7 and Figure 8. Additionally, the linear and asymptotic relations (Equation (20)) are included in the figures to show the correlation with the initial linear response and almost linear hardening behaviour. Generally, very good compatibility between both predictions and experiments can be observed. Nevertheless, the relationships from Equation (13) seem to better fit the experimental data.

The proposed moment–curvature relations from Equations (13) and (28) can be effectively applied to the prediction of the moment capacity and the curvature capacity (or rotation capacity) for limit design purposes. The curvature capacity can be directly calculated from the stress–strain curve and sectional dimensions $\kappa_{U}=\varepsilon_{U}/H$, and then the moment capacity $M_{U}$ is calculated from Equation (13) or Equation (28). Results of model predictions are presented in Table 4, showing very good consistency with the experimental data $M_{Exp}$ for beams H50 × 95 × 10.5 and H70 × 120 × 10.5 bent about the major axis. A higher discrepancy is observed for beams H95 × 50 × 10.5 and H120 × 70 × 10.5 under flexure about the minor axis. Comparing the results from Table 4 for $\kappa_{U}$ with the curvature ductility shown in Figure 6 and Figure 8 one can see high inconsistency. The same trend was observed in [29]. Thus, the moment capacity $M_{M}$ was additionally calculated for the reduced strain ductility $\kappa_{M}=\kappa_{U}/2$, which shows very good agreement with the experiments for beams bent about the minor axis. Such reduction is recommended in regulations [24] for all design cases, as described in Equation (9).

## 7. Comparisons to Three-Point Bending Tests

The sectional moment–curvature relation in Equation (28) can be used to solve boundary-value problems of beams. Substitution of Equation (11) into Equation (28) results in the following displacement equation:

(32) $$E\left(J_{R}-J_{H}\right)w^{\prime \prime }\left(x\right)+\frac{\left(E_{0}-E\right)\left(J_{R}-J_{H}\right)\kappa_{0}w^{\prime \prime }\left(x\right)}{\sqrt[{2k}]{\kappa_{0}^{2k}+{\left\{{\left[w^{\prime \prime }\left(x\right)\right]}^{2}\right\}}^{k}}}+M\left(x\right)=0.$$

A three-point bending test (B3), shown in Figure 3b, is selected since the experimental curves of maximum moment versus end-rotation are available in [29]. For the regarded beams, the moment function is $M\left(x\right)=P\left(L-x\right)$, and the boundary conditions for the displacement and slope (angle of rotation) are the following: $w\left(L\right)=0$, $w^{\prime }\left(0\right)=0$. The *NDSolve* procedure of *Wolfram Mathematica* software was used to obtain a numerical solution to differential Equation (32) for four beams with different sets of material parameters.

**Figure 9:**
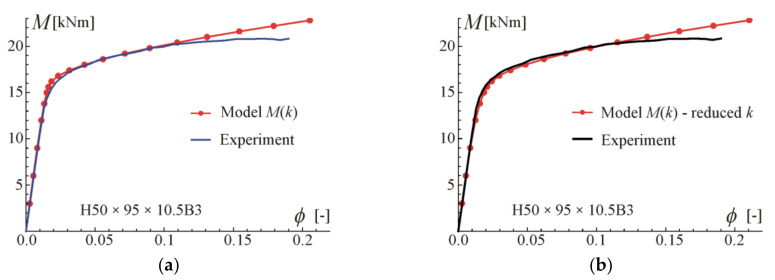
Maximum moment-rotation curves and comparison with experimental data of [29] for two exponents: (**a**) based on fit k=1.89, (**b**) for reduced k=1.1.

**Figure 10:**
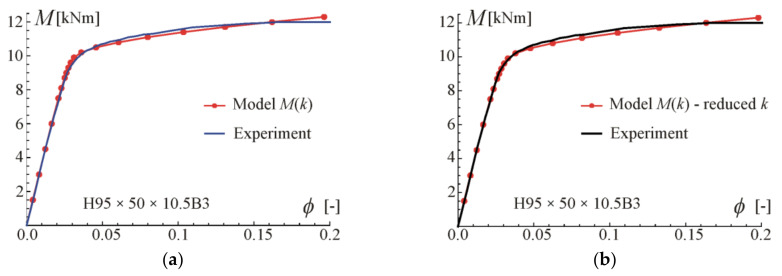
Maximum moment-rotation curves and comparison with experimental data of [29] for exponent: (**a**) based on fit k=2.14, (**b**) for reduced k=1.8.

**Figure 11:**
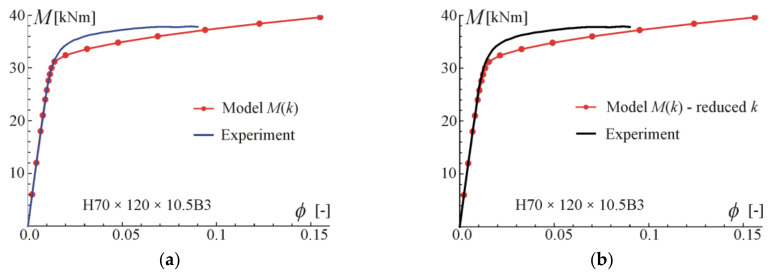
Maximum moment-rotation curves and comparison with experimental data of [29] for two exponents: (**a**) based on fit k=2.05, (**b**) for reduced k=1.6.

**Figure 12:**
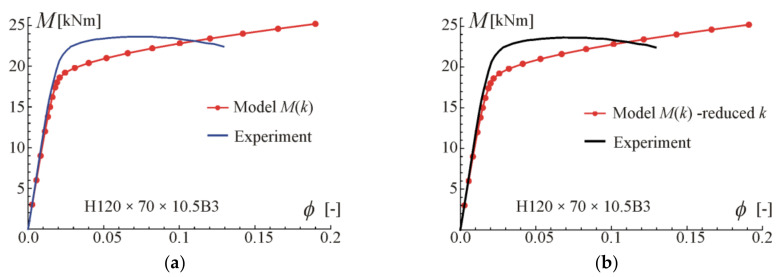
Maximum moment-rotation curves and comparison with experimental data of [29] for two exponents: (**a**) based on fit *k* = 1.84, (**b**) for reduced *k* = 1.6.

Comparisons of moment-rotation curves with experimental data given in [29] are presented in Figure 9, Figure 10, Figure 11 and Figure 12. In every considered case, the calculations were conducted up to the point where the maximum (experimental) value of bending moment or rotation angle was reached. The maximum value was assumed as given on the moment–curvature curves. This can differ from the extreme value on the moment-rotation angle curves. Predictions of the model described by Equation (28) are obtained for parameters given in Table 2 (four-point bending beam calibration) with exponent value $k\left(n\right)$, and additionally with reduced values of *k* to show the influence of this parameter and possibly to better fit. In the carried out calculations, calibration results from Table 2 were used since they give the best fit to the experimental data.

For beams H50 × 95 × 10.5B3 and H95 × 50 × 10.5B3, very good compatibility between predictions and tests can be observed. It is shown that by reducing *k*, better consistency with experiments can be obtained. The prediction curves are obtained for the perfect point load of the beam from Figure 3b, while in the tests the load forces were transferred to the upper flanges through stiffening steel plates to prevent web crippling, so the load is somehow distributed on some subregions of the beam. According to experimental data given in [29], the ultimate moment in B3 tests was larger by 18% (on average) than for B4 tests of beams made of the same material and cross-section. In the authors’ opinion, the way of load application is the major source of differences in the moment capacity and in the knee region on the plotted graphs. In the case of beams H70 × 120 × 10.5B3 and H120 × 70 × 10.5B3, a higher discrepancy between predictions and experiments can be observed. However, the results given in Figure 7 and Figure 8 for four-point bending beams show very good agreement with experiments for the same material and beam cross-sections. Those differences are probably due to scatter in material properties.

Next, verification of the proposed model is carried out for three-point bending beams when values of parameters are taken from calibration of the stress–strain relationship in Equation (3). Two options are investigated; one with the usage of formulae according to Equation (8) and another with the usage of Equation (9). The major difference between the variants lies in the value of hardening modulus E. The initial modulus $E_{0}$ was assumed the same for all analysed cases. Numerical values of the obtained parameters are given in Table 5 for the procedure according to Equation (8), and in Table 6 when Equation (9) is used. In both cases, the reduced value of exponent *k* was used.

**Figure 13:**
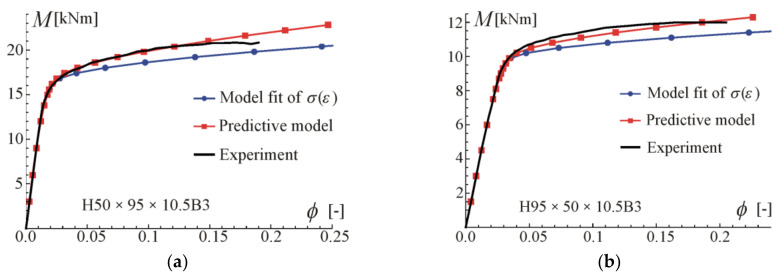
Prediction of maximum moment-rotation curves and comparison with experimental data of [29] for beams: (**a**) H50 × 95 × 10.5B3, (**b**) H95 × 50 × 10.5B3.

**Figure 14:**
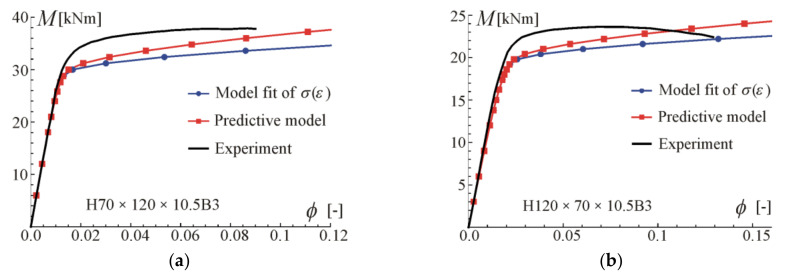
Prediction of maximum moment-rotation curves and comparison with experimental data of [29] for beams: (**a**) H70 × 120 × 10.5B3, (**b**) H120 × 70 × 10.5B3.

Comparisons of moment-rotation curves with experimental data are presented in Figure 13 and Figure 14. For beams H50 × 95 × 10.5B3 and H95 × 50 × 10.5B3, very good compatibility between predictions and experiments can be observed when calibration Equation (9) is used. Note that prediction with the usage of formulae from Equation (8) is conservative. Similar conclusions can be drawn in the case of beams H70 × 120 × 10.5B3 and H120 × 70 × 10.5B3.

## 8. Summary and Conclusions

Low-hardening aluminium alloy grades 6061-T6, and 6082-T6, and 7075-T6 are characterised by a rounded stress–strain relationship, which is close to a linear elastic-plastic hardening behaviour. In this paper, the alloys’ characteristics are described by Richard–Abbott type relationships expressing stress explicitly in terms of strain. The choice of the stress–strain curve format is essential to the derivation of the relationship between moment and curvature for slender beams. We use the RA type constitutive relationship $\sigma \left(\varepsilon \right)$, which allows us to obtain a new analytical form of $M\left(\kappa \right)$. In contrast, usage of RO format $\varepsilon \left(\sigma \right)$ for constitutive relationship necessarily requires numerical integration to establish a series of moment–curvature points, which can then be approximated to obtain a function form for the moment–curvature relation [35].

Based on the regarded format of constitutive relation $\sigma \left(\varepsilon \right)$, an original sectional relation of moment as an explicit function of curvature for beams is derived in this paper. In total, we have proposed two moment–curvature equations: the first one directly based on the $\sigma \left(\varepsilon \right)$ relationship, which is quite complex but precise, and another, which is its close approximation. In the first case, the obtained expression contains special functions, so we introduced a second simplified moment–curvature relationship. The definition of the second constitutive equation adopts characteristic parameters from the more complex model, and a conversion formula for exponents is established to have a high level of equivalency between both descriptions. The novel simplified moment–curvature relation is more useful for practical purposes of solving boundary-value problems, which is confirmed by several comparisons to experimental data. The predictive capabilities of the model are verified on beams in four-point and three-point bending tests.

Since the proposed novel moment–curvature relations are based on the direct approximation of the stress–strain curve, design methods such as the CSM can be improved. A full-range and continuous relation described by a single mathematical formula accounts for curvature in the knee transition region of the stress–strain curve. Accounting for this feature makes it possible to improve the determination of parameters needed for the design of statically indeterminate beams according to the limit state approach.

The introduced moment–curvature relations can be effectively applied to the prediction of the ultimate moment and the curvature capacity (or rotation capacity) for limit design purposes. Since the nonlinear relations closely reproduce experimental curves, they can also be used for the determination of beam deflections in serviceability limit state design. In such applications, the reliable determination of two material parameters is crucial, namely estimating or calculating the hardening modulus E and the exponents n or *k*. Moreover, the influence of the specific manner of load application to the stocky RHS beam structures is identified but requires further investigation. Despite the presented utility of the proposed models, further research is needed to formulate reliable recommendations for the calculation of the model parameters. Although the proposed models are applied to selected aluminium alloys, they can be successfully used for other metallic alloys exhibiting similar stress–strain responses, including high strength steels, stainless steels, and cold-formed steel products.

## Figures and Tables

**Table 1:** Values of exponent *k* for several values of *n*.

***n***	0.75	1.00	1.25	1.50	1.75	2.00	2.25	2.50	2.75	3.00	3.25	3.50	3.75
***k***	0.72	0.94	1.15	1.34	1.52	1.69	1.84	1.99	2.12	2.24	2.35	2.45	2.54

**Table 2:** Curve fit results for Equation (13) with additional parameters.

Beam [B4]	*E*_0_ [GPa]	*E* [MPa]	*ε*_0_ [-]	*n* [-]	*k*(*n*) [-]	*κ_E_* [m^−1^]	*κ*_0_ [m^−1^]	*M*_0_ [kNm]
H50 × 95 × 10.5	67.7	700	0.00331	2.89	1.89	0.0699	0.0936	16.1
H95 × 50 × 10.5	67.6	557	0.00341	2.79	2.14	0.138	0.179	9.87
H70 × 120 × 10.5	67.9	356	0.00351	3.18	2.05	0.0585	0.0756	30.8
H120 × 70 × 10.5	68.0	696	0.00315	2.17	1.84	0.0903	0.112	18.5
Average	67.8	557	0.00334	2.76	1.98	-	-	-

**Table 3:** Curve fit results for Equation (28) with additional parameters.

Beam [B4]	*E*_0_ [GPa]	*E* [MPa]	*ε*_0_ [-]	*k* [-]	*κ_E_* [m^−1^]	*κ*_0_ [m^−1^]	*M*_0_ [kNm]
H50 × 95 × 10.5	69.9	801	0.00316	1.76	0.0667	0.0892	15.8
H95 × 50 × 10.5	68.5	674	0.00333	2.10	0.134	0.175	9.76
H70 × 120 × 10.5	70.0	438	0.00336	1.89	0.0560	0.0724	30.4
H120 × 70 × 10.5	68.6	841	0.00308	1.84	0.0882	0.110	18.3
Average	69.2	688	0.00323	1.90	-	-	-

**Table 4:** Predicted ultimate moment and curvature from Equations (13) and (28).

Beam [B4]	*ε_U_* [-]	*κ_U_* [m^−1^]	*M_U_* (13) [kNm]	*M_U_* (28) [kNm]	*M_M_* (28) [kNm]	*M_Exp_* [kNm]
H50 × 95 × 10.5	0.06	1.267	18.16	18.22	16.93	18.04
H95 × 50 × 10.5	0.06	2.419	10.88	10.99	10.33	10.35
H70 × 120 × 10.5	0.06	1.002	32.80	32.85	31.66	33.00
H120 × 70 × 10.5	0.06	1.719	21.22	21.54	19.79	19.66

**Table 5:** Parameters obtained for Equation (28) according to Equation (8).

Beam [B3]	*E*_0_ [GPa]	*E* [MPa]	*ε*_0_ [-]	*k* [-]	*κ_E_* [m^−1^]	*κ*_0_ [m^−1^]	*M*_0_ [kNm]
H50 × 95 × 10.5	68.0	217	0.00338	1.1	0.0714	0.0956	16.4
H95 × 50 × 10.5	68.0	217	0.00338	1.1	0.136	0.178	9.83
H70 × 120 × 10.5	68.0	200	0.00333	1.6	0.0556	0.0719	29.2
H120 × 70 × 10.5	68.0	200	0.00333	1.6	0.0955	0.119	19.4

**Table 6:** Parameters obtained for Equation (28) according to Equation (9).

Beam [B3]	*E*_0_ [GPa]	*E* [MPa]	*ε*_0_ [-]	*k* [-]	*κ_E_* [m^−1^]	*κ*_0_ [m^−1^]	*M*_0_ [kNm]
H50 × 95 × 10.5	68.0	476	0.00339	1.1	0.0717	0.0960	16.5
H95 × 50 × 10.5	68.0	476	0.00339	1.1	0.137	0.178	9.86
H70 × 120 × 10.5	68.0	437	0.00335	1.6	0.0558	0.0721	29.3
H120 × 70 × 10.5	68.0	437	0.00335	1.6	0.0958	0.119	19.5

## Data Availability

Data are contained within the article.
